# A mixed-method analysis of the contribution of informal sport to public health in Australia

**DOI:** 10.1093/heapro/daae048

**Published:** 2024-05-29

**Authors:** Ruth Jeanes, Justen O’Connor, Dawn Penney, Ramon Spaaij, Jonathan Magee, Eibhlish O’ Hara, Lisa Lymbery

**Affiliations:** School of Curriculum Teaching and Inclusive Education, Faculty of Education, Monash University, McMahon’s Road, Frankston 3199, Victoria, Australia; School of Curriculum Teaching and Inclusive Education, Faculty of Education, Monash University, McMahon’s Road, Frankston 3199, Victoria, Australia; School of Curriculum Teaching and Inclusive Education, Faculty of Education, Monash University, McMahon’s Road, Frankston 3199, Victoria, Australia; School of Education, Edith Cowan University, 270 Joondalup Drive Joondalup, WA 6027, Australia; College of Sport, Health and Engineering, Victoria University, Nicholson & Buckley Streets, Footscray, 3011, Victoria, Australia; School of Governance, Utrecht University, Bijlhouwerstraat 6 3511 ZC, Utrecht, The Netherlands; School of Curriculum Teaching and Inclusive Education, Faculty of Education, Monash University, McMahon’s Road, Frankston 3199, Victoria, Australia; School of Education, Edith Cowan University, 270 Joondalup Drive Joondalup, WA 6027, Australia; School of Education, Edith Cowan University, 270 Joondalup Drive Joondalup, WA 6027, Australia; College of Sport, Health and Engineering, Victoria University, Nicholson & Buckley Streets, Footscray, 3011, Victoria, Australia

**Keywords:** informal sport, sport participation, community health promotion, health inequalities

## Abstract

Informal sport is a growth area of sport participation but there has been limited examination of how informal and unstructured forms of participation may contribute to health outcomes that are important for public health. This article aims to address the current lack of data examining the health outcomes associated with informal sport participation and consider the potential role of informal sport within efforts to promote healthier communities through sport. The article seeks to broaden understanding of how informal sport participation can contribute to health outcomes, particularly with regard to increasing physical activity and enhancing mental health and social connection. The article discusses the findings of an Australian mixed-method study that draws on observation, survey, interview and focus group data to examine the prospective health and social benefits of informal sport participation for adults. The findings demonstrate that informal sport participation can contribute to physical and mental health outcomes and facilitate social connection. Analysis of the observation data enabled an examination of the economic value of informal sport in relation to the health benefits it affords. The study provides valuable evidence of the value of informal sport for enhancing community health and broadens understanding of how sport can be utilized as a health promotion resource. The article concludes by suggesting that through leveraging existing infrastructure and the self-organizing aspects of informal sport, local government and health stakeholders can harness its potential to improve public health outcomes and address health inequities.

**Contribution to Health Promotion**

- The article demonstrates the physical, social and mental health benefits that can occur from informal sport participation.
- Physical inactivity is a significant issue for health promotion. This research illustrates that informal sport can support health promotion efforts to address physical inactivity within communities.
- The article argues that by leveraging existing infrastructure and the self-organizing aspects of informal sport, local government and health stakeholders can harness its potential to improve public health outcomes and address health inequities.

## INTRODUCTION

Community club-based sport is recognized as a valuable health promotion resource that can contribute to enhanced mental and physical health, social connection and social cohesion (Davies *et al*., 2021). Increasing participation in sport has been a long-term objective of Australian federal government policy, however, in recent years adult participation in club-based sport has plateaued in many sports and declined in others (Eime *et al*., 2016). This trend is not unique to Australia and has been noted in other Global North countries including New Zealand, Canada and England (Gilchrist and Wheaton, 2017). Australian data suggest that 82% of the population over 19 years of age do not participate in club-based sport (Eime *et al*., 2019). Despite low participation rates, the federal government, via the Australian Sports Commission and national governing bodies of sport, continues to invest substantial public funds in supporting club-based participation, often justified by the contributions that it can make to enhancing public health (Stewart *et al.*, 2004). Evidence would suggest that the ability of club-based sport to promote physical, mental and social health is limited to a narrow proportion of the population playing sport in this way (Weed, 2016).

While participation in club-based sport is contracting there has been a growth in informal, unstructured and casual forms of sport participation (Cameron, *et al.*, 2022). Informal sport participation refers to participation that takes places outside of the formal affiliated structure of sport (Wheaton and O’Loughlin, 2017; Jeanes *et al*., 2019). Informal sport is a growing area of sport participation, but there has been limited examination of the contribution that this format can make to enhancing community health (Kokko, 2014). This article utilizes a mixed-methods approach that draws on observation, survey, interview and focus group data to examine the prospective health and social benefits of informal sport participation for individuals and communities. In doing so, the article seeks to broaden understanding of how sport participation can promote health, particularly with regard to increasing physical activity, enhancing mental health and enhancing social connection. This article aims to address the current lack of data examining the health outcomes that informal sport can contribute to and explore the potential economic value of informal sport participation within communities. The article critically considers the potential of informal sport as a context to support public health outcomes. The article concludes that informal sport is currently a largely untapped resource within health promotion policy and that if sport is to make a meaningful contribution to population-wide health outcomes, informal formats need to be supported within communities and accorded greater priority in funding and policy frameworks.

## BACKGROUND

### Physical activity, sport and health promotion

Australia, like many countries globally, is a relatively sedentary nation with 75% of adults not physically active enough to accrue health benefits (ABS, 2022). Physical inactivity contributes to a number of health-related issues including overweight and obesity, increased risk of coronary heart disease and increased risk of certain cancers (King, 2012; Pinto *et al*., 2020; Katzmarzyk *et al*., 2022). Well-established health inequities leave certain populations at greater risk of physical inactivity and impacts on their physical health. Adults from low socioeconomic, multicultural or Indigenous backgrounds and people with disabilities are likely to be less physically active than the wider population and also more vulnerable to ill health (Gordon-Larsen *et al*., 2006; Newman *et al*., 2015).

The annual cost of physical inactivity globally is estimated to be US$47.6 billion (Santos, *et al*., 2023). In Australia alone, the estimated cost to the healthcare system caused by physical inactivity is AUD$2.4 billion per year (Australian Institute of Health & Welfare, 2023). In Australia, a risk factor impact (RFI) model developed by Deakin Health Economics (Cadilhac *et al*., 2011) suggests annual healthcare cost savings of up to AUD$300 for each person who becomes physically active.

Alongside physical health issues, and again reflecting global trends, mental ill health is a significant challenge impacting the Australian population. Globally, WHO data suggest that one in eight adults are living with mental illness (WHO, 2019), with mental ill health more prevalent in countries such as the UK where one in four adults experience a mental illness in any given year (Statista, 2022). In Australia, one in five adults experience a mental illness in any given year (AIHW, 2022). Mental ill health similarly places a substantial economic burden on the economy estimated to be USD2.5 trillion annually at a global level (Arias *et al*., 2022).

There is an established literature base that suggests participation in sport can contribute to various health benefits, with these largely tied to sports capacity to increase people’s involvement in physical activity. Physical benefits associated with sport participation include reduced risk of cardiovascular disease, developing type 2 diabetes and some cancers (Warburton and Bredin, 2017). Participation in sport is recognized to provide mental health benefits including contributing to protective factors in relation to anxiety and depression (Panza *et al*., 2020). Furthermore, existing studies point to the capacity of sport to facilitate social connection and feelings of belonging which have been demonstrated to contribute to both physical and mental health (Walseth, 2006; Hoye *et al*., 2015). Recent studies utilizing a social return on investment (SROI) methodology, suggest participation in organized sport contributed to an estimated AUD$1.8 billion in mental health benefits, AUD$1.20 billion in enhanced social capital and AUD$1.00 billion in health-related quality of life (SportWest, 2021). The health benefits sport can provide therefore have significant economic implications for communities; however, levels of club-based sport participation remain low across the adult population.

### Informal sport and health promotion

Academic analysis of informal sport participation has been expanding in recent years particularly focusing on action and lifestyle sports such as skateboarding, parkour and surfing (Wheaton and Doidge, 2015). There has been limited exploration of informal formats of more traditional or mainstream sports (such as soccer, basketball, cricket) and studies that have been undertaken have not engaged significantly with the wider health promotion potential of informal sport. Analysis to date has illustrated some of the policy and governance challenges that exist in legitimizing informal sport within the sport participation landscape (Wheaton and O’Loughlin, 2017; Jeanes *et al*., 2019), issues surrounding access, facilities and space for informal participants (Aquino *et al*., 2022; Jeanes *et al.*, 2022) and the role of informal sport in migrant and refugee resettlement (Thorpe and Wheaton, 2021; Spaaij *et al*., 2023).

The context most extensively explored when examining informal sport activity and health and wellbeing benefits is Parkrun, the global phenomenon where volunteers organize a 5-km free community, walk, jog or run every Saturday morning. Since its inception in 2011, there are 2000 locations in 22 countries that host a regular Parkrun event. In Australia, Parkruns are held at 473 locations with 930,000 participants. In 2016, private health insurance company Medibank became a major sponsor of Parkrun Australia signaling the links between health and this form of informal sport participation. Studies examining Parkrun have identified its capacity to initiate and maintain health-enhancing behaviors including physical activity (Stevinson *et al*., 2015) by providing a flexible, open and low-cost activity that offers a regular structure for participation. Grunseit *et al.* (2020) suggest that Parkrun is effective at engaging traditionally underrepresented populations in physical activity and is a flexible model that suits the needs of participants to engage in physical activity at a level that suits them. The authors suggest that although a relatively small evidence base is available, Parkrun can positively impact participant’s health and wellbeing. This is echoed by Quirk et al. (2021), who concluded from a survey of 60,000 participants that Parkrun was effective at increasing physical activity levels among respondents from socioeconomically deprived areas. The findings point to the potentially important contribution informal sport such as Parkrun can make to public health and in addressing inequalities that arise from a lack of access to affordable physical activity opportunities.

As outlined above, there has been a rich array of studies examining the impact of physical activity on various health outcomes (Warburton and Bredin, 2017). More specifically, the potential of sport as a health promotion resource has been recognized, however, the substantive body of research available has largely focused on traditional, structured sports clubs as the context of the study (Geidne *et al*., 2019; Robertson *et al.*, 2019; O’Connor *et al*., 2022). Informal sport is a form of participation that sits between existing physical activity studies and those conducted within structured, club-based sporting contexts. Despite the growth in informal sport participation, exploration of the health promotion potential of informal sport has been largely restricted to Parkrun as one context of informal activity. This article seeks to contribute to an evidence base examining the health, social and economic benefits of informal sport to participants.

## METHODS

### Defining informal sport in the context of the study

Informal sport is a contested concept with various terminologies (e.g. lifestyle, action, pick-up, social sport) that are often used interchangeably under the umbrella of informal participation. In this project, we define informal sport participants as individuals and groups who meet all of the following three criteria:

1. Are not committed members either financially or to a schedule/competition of a formal competitive sporting club, organization or governing body;
2. Organize their regular participation in sport outside of traditional structures;
3. Participate in activities recognizable as sports like soccer, basketball, cricket, volleyball, swimming, running and cycling (or modified versions of these), rather than activities more typically associated with fitness or leisure such as aerobics, yoga or personal training.

### Study design

The data discussed in this article were collected as part of a 3-year study examining informal sport as a health and social resource. The study utilized a mixed-method approach with data collection occurring in two states within Australia (Victoria and Western Australia), four local government areas (2 from each state) and 16 locations (4 from each local government area). The data discussed in this article arise from observational, survey, interview and focus group methods. Observation comprised an adaptation of the Systems for Observing Play and Recreation in Communities (SOPARC) protocol (McKenzie *et al*., 2006; McKenzie and van der Mars, 2015) to capture details of participants and the type and level of participation, alongside the descriptive data captured on participation via SOPARC, these data were also utilized to undertake an analysis examining the economic contribution of physical activity resulting from informal sport participation. A survey was used to explore social connection, and focus groups and individual interviews with informal sport participants enabled in-depth analysis of participant experiences and perceived impacts on health and wellbeing. As outlined, the aim of the study was to develop an evidence base outlining the potential health contributions of informal sport. A mixed-method approach was appropriate for collating multiple perspectives and sources of information examining the value of informal sport, ranging from exploring participant experience to quantitative data examining participation. Each of these methods, the respective participant samples and analysis techniques are discussed further in turn.

### Observation data

Unlike formal sport, a lack of record keeping and membership data meant one of the best ways to capture informal sport participation was to observe it in situ. To capture informal sport participation in community settings, the SOPARC instrument was deployed based largely on the procedures outlined by McKenzie *et al.*, (2006). Systematic observation provided an opportunity to directly observe and record simultaneously the physical environment and the sporting context while capturing objective data about participation with strong internal validity (McKenzie and van der Mars, 2015). The SOPARC utilizes momentary time sampling techniques whereby systematic scans of individuals undertaking their activities within pre-defined target areas at pre-determined time periods are made. Data were summarized to describe the number of participants by activity, mode (informal) and intensity (METS), within estimated age and gender groupings. Validity of the activity codes used by SOPARC has been established through heart rate monitoring (McKenzie *et al*., 1991, 2006) and accelerometry (Ridgers *et al*., 2010).

Following familiarization training using videos, observers from each state collectively completed field-based training and initial reliability checking. Additional inter-rater reliability data were periodically collected during the observation period and achieved the target of > 80% agreement. Identification and selection of sites for observation occurred in consultation with local government officers. To be considered for inclusion, areas needed to contain suitable open park space and have facilities or equipment that might support informal sport participation. The four sites in each local government area were selected based on the following criteria:

1. Recommended by council officers as sites of interest for informal sport.
2. Suitably close to housing with good community access.
3. Provided formal sporting facilities and/or open spaces suitable for informal participation (open grassed areas, basketball court rings).
4. Sufficient space for multiple participant groups/ types.
5. Ranked by researchers as viable sites for observations and provided a diversity of site contexts within and across the local government areas.

Settings included those that comprised official sporting fields, sporting courts or boundaries and were used by formal sporting competitions, and more residential park spaces with modified facilities but no formal sport organization use. Sites were more conducive to team-based sports than activities like cycling, swimming and running; the exceptions were Scarborough Beach in WA and to a much lesser extent Carlisle Park in Victoria. Observations also extended to indoor multi-court facilities adjacent to open space at two of the sites in WA. The equivalent of 1 full week’s worth of morning and evening observations took place at each site across a 2-week period.

The intended purpose of SOPARC is to capture all forms of activity in community spaces. Our primary focus was to capture informal sport participation with a secondary purpose of capturing formal or official sport participation as a point of comparison. Observers recorded sport as informal when it was clear there was no organizing agent (club personnel, coach, structure) and where this was ambiguous, participants were asked if they were participating as members of a sporting club. Activities excluded from being counted as either formal or informal sport included walking, spectating, yoga, strength exercise, fitness station, group fitness, dance, climbing/parkour, kite surfing, stand-up paddle boarding and aqua aerobics. There were observations of semi-organized informal sport, particularly in indoor settings. These involved an organizing agent and payment of court-hire fees, but participants were not members of a club and were therefore considered as informal participation within observations.

Data from paper-based forms were manually entered into Microsoft Excel at the end of a collection period and this was collated into SPSS V.28 for analysis. We report the findings for the busiest 30-min interval within any 1-hr observation period. Invariably, this coincided with the second half of the 1-hr observation window in the mornings and evenings.

Physical activity benefits for informal sport were accounted for by calculating MET-hours gained. A MET represents the ratio of energy expended divided by resting energy expenditure. MET-hours gained represents the MET-hours per person per day gained as a result of sport participation. One MET is defined as the energy it takes to sit quietly, equivalent to a caloric consumption of 1 kcal/kg/hr. For example, if jogging (seven METs) is done for 1 hr, it results in seven MET-hours. This means the person has expended energy equivalent to 7 times what they would have expended resting for the same duration. Two different approaches were used to calculate an estimate of energy expenditure. One relied on estimates from the SOPARC observations whereby participants during scans were coded as sedentary, walking or very active (later converted to METs). The other involved using the Compendium of Physical Activity (2011) to later apply MET values based on the sporting activities observed. The Compendium provides a comprehensive list of physical activities and their validated MET values (Ainsworth *et al*., 2000, 2011). MET-hours gained were derived by multiplying the METs associated with the type and intensity of the activity by the time spent performing the activity using hours as the unit of analysis.

The observed MET-hours gained using the SOPARC tool were typically lower than the intensities recorded in the Compendium (see Table 1), particularly for formal sport. This may have been due to an overestimation of the Compendium values, given not all formal sport we witnessed was at the intensity of full match play, and an underestimation on behalf of the SOPARC estimates due to the timing of the single point activity scan. As a result, the mean value of METS from both the compendium and observed estimates was used in the final calculation as a representation.

**Table 1:** Relative METS of each activity

	Formal sport mets		Informal sport mets	
**Sport**	**CPA** ^*^	**SOPARC** Mean (SD)	**CPA** ^#^	**SOPARC** Mean (SD)
Jog/Run	10.00	6.00 (0.00)	7.00	5.41 (1.27)
Cycling	10.00	–	6.80	4.67 (1.72)
Skateboard	6.00	–	5.00	4.18 (2.05)
Soccer	10.00	3.91(1.96)	7.00	3.75 (1.92)
Cricket	4.80	2.72 (1.61)	4.80	2.92 (1.78)
Basketball	8.00	4.92 (1.7)	4.50	4.26 (1.90)
Tennis	7.30	–	5.00	2.63 (1.71)
Netball	*7.80*	3.18 (1.82)	*5.00*	2.70 (1.04)
Hockey	7.80	3.57 (1.97)	*5.00*	2.25 (1.84)
Volleyball	8.00	–	*5.00*	3.24 (1.99)
AFL	*10.00*	3.38 (1.92)	*7.00*	3.88 (1.97)
Rugby	7.80	–	6.30	3.21 (1.35)
T-Ball	5.80	2.19 (1.48)	5.80	–
Softball	5.00	2.60 (0.91)	5.00	–
W’Chair BBall	7.80	4.03 (2.10)	7.80	–
Badminton	7.00	4.80 (1.65)	5.50	3.54 (1.71)
Swimming	6.00	5.18 (1.51)	6.00	5.44 (1.28)

^*^Based upon the competitive or sporting version found in the compendium of physical activity.

^#^Based upon the casual, recreational version found in the compendium of physical activity.

*Italics*: Not in the compendium but based on similar activities.

A number of assumptions were made in the calculation of MET-hours gained. These assumptions were necessary as individual identity/histories were not collected. For comparison purposes, the same assumptions were applied to formal sport participation. Assumptions consisted of the following:

- If people were not participating in informal sport as observed, they would not be doing any alternate physical activity. Consequently, MET-hours calculated here are MET-hours gained above being sedentary.
- The MET-hours gained is not a one-off, rather it represents a consistent pattern of behavior.
- Each data point represents a unique person participating once per week rather than being the same person on multiple occasions.
- Each person is participating in the activity for a minimum of 60 min.

Only data from those estimated to be over 17 years of age were included. This reflected that our project was particularly directed towards youth and adult participation.

### Economic analysis of observational data

Having identified the total MET-hours gained across the equivalent of a 1-week observation period, MET-hours gained per day, per person was calculated. The RFI model developed by Deakin Health Economics in Australia (Cadilhac *et al*., 2011), conservatively estimates transitioning one person from inactive to active status produces an average savings of $300 per person per year over their lifetime (3.8% of the total healthcare cost—comparable to US estimates). The minimum recommendation for physical activity translates to 390 MET-hours per year for adults (Wu *et al*., 2011) meaning the contribution of each MET-hour of physical activity equates to $1.30. To make cost comparisons, Wu *et al*. (2011) suggest standardizing the duration of the intervention to 12 months for a potential 10,000 target population. Benefit in this hypothetical year was calculated as MET-hours gained per person per day multiplied by 1-year duration and then by 10,000.

### Social connection data collection

To understand levels of social connection among informal sport participants, an online survey was distributed via social media. The survey was distributed via Twitter (now X) through the research team and contacts in sports administration, the social media accounts of informal groups connected with as part of the research, through local council and sports governing bodies’ Facebook pages and Instagram accounts and social sport social media accounts. Social connection was measured using a modified version of the Social Connectedness Scale, initially developed by Lee and Robbins (1995). This modified version of the scale developed by Hoye *et al*. (2015) was adopted to allow comparison with their formal sport participation data. This measure comprised the following items: ‘I feel disconnected from the world around me’ (β = 0.770); ‘Even around people I know, I don’t feel that I really belong’ (β = 0.849); ‘I feel so distant from people’ (β = 0.882); and ‘I have no sense of togetherness with my peers’ (β = 0.835) generating a means score *M* = 20.16 (SD = 3.72; *n* = 1548) and Cronbach’s alpha of 0.90. For the same items, our sample (*n* = 109) returned a mean score *M* = 19.09 (SD = 4.34) and Cronbach’s alpha of.84 with inter-item correlations between 0.48 and 0.68.

A total of 153 participants accessed the survey with 109 participants completing it (71.2%). 28% percent identified as coming from an Australian cultural background, 23% as European, 48% represented a range of cultural backgrounds, while 16% did not answer. Age in years ranged from 18 to 67 with a mean age of 35.6 years (SD = 11.2). Just over half (51.4%, *n* = 55) participated in only informal sport, 21.5% (*n* = 23) participated in a combination of formal and informal sport, 18.7% (*n* = 20) participated in only formal sport, while 8.4% (*n* = 9) indicated they participated in fitness-focused activities (yoga, exercise classes). The sample comprised 60% identifying as male, 39% identifying as female and 1% non-identifying, with 90% of formal sport participants and 62% of informal participants indicating they were male.

### Interviews and focus groups

Semistructured interviews were conducted with informal sport group leaders. Leaders were defined as individuals who organized and managed informal sport groups, they were usually the point of contact for participants, determined the location and timing of gathering and usually promoted the group through social media. Interviews were conducted with 42 informal sport group leaders. The research team connected with leaders via the observations, with groups approached and invited to take part. Leaders were also connected via a snowballing approach with recommendations from those interviewed of other groups to contact. Six of the leaders were women and 36 were from non-Caucasian background. The leaders were responsible for organizing groups participating in a number of different sports including badminton, cricket, basketball, skate, BMX, tennis, volleyball. Interviews focused on examining motivations for informal sport participation, challenges and facilitators to participation and experiences of participating including impacts on health and social relationships. These areas were considered in relation to individual’s personal experiences and what they observed across group members. Interviews were transcribed in full and thematically analysed (Joffe, 2011). Inductive analysis was initially undertaken to identify key themes which were then used to develop a coding framework that was developed from emergent themes. Analysis was initially undertaken independently by four members of the research team to develop the coding framework. Once the framework was agreed upon, the researchers coded five transcripts and compared for similarity, with a 90% similarity rating achieved.

Focus Groups were undertaken with a further 62 informal sport participants with 12 informal groups taking part in the focus groups. We had observed these participants during our SOPARC data collection and they indicated a willingness to be involved in focus groups. A total of 39 participants in the focus groups identified as men, 23 identified as women. Nine focus groups consisted of men-only participants, three women-only. Ninety percent were non-Caucasian. Four groups consisted of participants from predominantly Indian heritage (three were cricket groups, one soccer), three with participants of Afghan heritage (two soccer groups, one Sangarag, a traditional cultural sport), two groups of participants from Turkish heritage (two soccer), one group of participants from Kenyan heritage (Soccer group) and one of participants from South East Asia (badminton). The group of participants from mixed cultural background participated in futsal.

The focus group questions were similar to those used for individual interviews, with the group discussions providing greater opportunity for reflection and discussion by participants. The analysis process used mirrored the interview analysis, with the researchers independently analysing three focus group transcripts and identifying key themes, these were compared to the interviews with similar themes emerging. The coding framework utilized for the interviews was subsequently used to code the focus group transcripts with a further layer of analysis examining comparisons between leader and group experiences of participation.

While each aspect of data collection was initially analysed as an independent data set, aspects of triangulation occurred where appropriate. For example, participants recounting how they perceived participation impacted on the level of fitness was considered alongside observational data and findings of the survey data relating to social connection were also considered alongside detailed accounts from participant focus groups and interviews.

### Ethics

The study received approval through the first author’s university ethics committee, project number 18504. Participants have been anonymized throughout the article.

## RESULTS

### Observation of informal sport: key findings

A total of 13,534 observations of people in various settings were made across the evaluation period. Of these, 7636 observations were associated with activities that could be connected to sport. More than double the observations were noted in Western Australia (WA) (*n* = 9247) compared to Victoria (*n* = 4287). Unlike Victoria, WA was not dramatically impacted by periods of COVID-19 public health orders that were in place at the time and the observation periods occurred at different times of the year due to the lockdown restrictions in Victoria.

Overall, informal sport including running, swimming and skateboarding represented 50.2% of the observations, while formal participation represented 49.8%. Informal sporting rates in Victoria were at 46% while in WA they were 52.9% compared to formal. The most popular sporting activities observed were cricket (26%), soccer (16.5%) and basketball (17.7%).

### Gender

Using a gender binary based upon observations as detailed in the SOPARC protocol (McKenzie *et al*., 2006) we estimated men (80%) were four times more likely to be playing informal sport than women (20%). If we combine all forms of activity, men were five times more likely to be observed than women across all sites.

### Observed metabolic equivalent of task (METs)

#### Economic contribution of informal sport through physical activity

Drawing on observational data we calculated that informal sport contributed 6646 MET-hours gained of physical activity across 16 sites in two states equating to a 1-week period for people estimated to be over 17 years of age. In the same period, informal sport contributed between 0.59 (mean MET intensity SOPARC) and 0.77 (Compendium of PA) MET-hours gained of physical activity per day per person compared to between 0.48 and 0.88 MET-hours gained per day per person for formal sport.

The minimum recommendation for physical activity translates to 390 MET-hours per year for adults (Wu *et al*., 2011). For people who would otherwise be sedentary, every MET-hour gained is worth approximately $1.30 for adults. At an average of 0.681 MET-hours gained per day per person, the informal sport we observed was comparable to formal sport (0.683) and a range of physical activity interventions aimed at increasing sedentary behavior, including individual behavior change adaptions (0.5), enhancing access to physical activity spaces (0.62), increasing social support (0.65) and community PA campaigns (0.44) (Wu *et al*., 2011). We observed approximately $8640 worth of informal sport physical activity in the equivalent of 1 week across the 16 settings, compared to $5219 for formal sport. For the equivalent of 10,000 people, this equates to a health saving of over AUD 1.62 million every 6 months. This excludes other benefits to be gained from social connection and the benefits detailed in the qualitative focus group data such as improvements in mental health.

### Social and mental health benefits of informal sport

#### Social connection

Social connection was measured quantitatively via an online survey and also discussed in greater depth during interviews and focus groups. Social connection is associated with enhanced mental and physical health and greater access to networks and resources (Holt-Lunstad, 2021). The survey data (*n* = 147) indicated there were no differences between the short-form measures of social connectedness across participation type (*F* (9, 93) = 1.30, *p* = 0.277). Using the 4-item Social Connectedness Scale (Lee & Robbins, 1995; Hoye, *et al*., 2015) informal participants reported mean = 18.37 (SD 4.0) and formal participants mean = 18.74 (SD 5.2). This suggests participants from this sample who play informal sport have comparative levels of social connection to people who play formal sport or a combination of informal and formal sport.

The importance of informal sport for fostering social connection was a key theme elaborated on during qualitative data collection. Informal groups supported participants to meet new people, establish friendships and build trusting bonds and relationships with other participants. As this group leader described,

*I think it’s just that social connection, I think probably that’s the most important thing. A lot of the women, some play, some have played, a lot of them haven’t played, they just come and have a go. But they really enjoy meeting other women and bonding. I think that bonding aspect is really valuable. (Women’s Soccer Group Leader)*

Participants in focus groups suggested that informal groups were safe spaces where they felt welcomed, valued and accepted. This leader explained,

*It [informal sport group] because most of us are immigrants, it gives..comfort that they’ve got someone in their corner to help them out when times are tough, or just to hang out as a stress relief, to go out and have a good time with people. (Cricket Group Leader)*

As discussed in the methodology, many of the focus group participants and group leaders were from multicultural communities and many of the participants discussed poor experiences in club-based sport, ranging from feelings of not belonging through to experiencing overt and institutionalized racism. Informal sport was ‘different’ in this respect for participants, with informal groups providing a setting that was responsive to their culture and beliefs and where they did not feel like outsiders. The perceptions of safety experienced within informal settings were important in supporting social connection. Informal sport groups were important to participants for facilitating a sense of community based on shared interests, cultural backgrounds and gender.

#### Mental health benefits

Participants discussed both the social and physical aspects of informal sport as contributing to their mental health. Many felt that informal sport offered them the opportunity to destress, be active in a supportive environment, and provide an escape from everyday stressors.

*I do come to the ground because of a couple of reasons: one of it is for the purpose of my mental health. You go to work and some of the jobs that we do is really taxing. It can really get into your head, so you need some – Because I work in the disability sector, and sometimes you go through hard shifts, so coming to the field, it helps your mental state. (Informal Futsal Participant)*

A number of the participants described how informal sport participation was an important mechanism for managing anxiety and depression arising from wider life circumstances.

*I have bad depression with spending many years on a bridging visa waiting to see if I can stay here. My soccer group has been constant through this. I go run around, we talk and joke and I feel better. I see them three, sometimes four times a week and it would be very hard without them. (Informal Sport Participant)*

One group leader described their informal group as being like ‘that Men’s Shed thing’ referring to the non-profit organization in Australia that provides space for men to gain social connection and improve their health. Reconvening and playing informal sport were particularly important in supporting the mental health of groups in Victoria after prolonged COVID lockdowns (262 days of lockdown over 2 years). This soccer participant explained,

*The benefits, like fitness is key, you get fitter and stuff, you get to free your head and mind from the pressure of work and COVID and everything that was worrying you. People were indoors for a very long time, you need to go out there and do something else. (Informal Soccer Participant)*

The fluidity and flexibility of groups enabled them to rebound quickly once lockdown restrictions were scaled back in Victoria with many groups returning to play as soon as government restrictions changed.

The perspectives of participants and leaders provide a rich account of the value and importance of informal sport groups in their lives.

## DISCUSSION

Collectively the findings of the study evidence and illustrate the health benefits associated with informal sport participation and the potential role that informal sport could have in health promotion efforts that utilize sport as a context. The accounts of participants point to the capacity of informal participation to foster similar benefits to those identified in studies examining the value of club-based sport within communities, including social connection, community connection alongside well-established mental and physical health benefits (Eime *et al*., 2013; Hoye *et al.*, 2015). Informal sport also offers a safe space (Spaaij and Schulenkorf, 2014) where participants feel accepted and included, often in ways they haven’t experienced in club-based sport settings.

The evidence suggests that informal sport can provide significant economic benefits in relation to health savings, and is comparatively cheaper to support than formal sport. Beyond the provision of facilities and infrastructure, which are often already available to support club-based participation, there is little additional investment or infrastructure required. Informal sport does not generate the same level of human resource investment needed as formal sport and requires functional, rather than ‘high-performance quality’, spaces and infrastructure. The apparent cost-effectiveness of informal sport, coupled with the positive contributions that we have detailed in this study suggest that it should be considered as a key resource in community health promotion work and in particular health promotion efforts that utilize community sport to achieve health outcomes such as increases in physical activity.

The data presented also point to informal sport’s value in terms of the participation reach it can achieve in comparison with more structured forms of community sport. Our qualitative data illustrate the capacity of informal sport to attract participants from multicultural communities who are often underrepresented in club-based setting (O’Driscoll *et al*., 2014). Informal sport therefore has a potentially valuable role to play in efforts related to a number of health and social policy objectives including social inclusion and physical activity promotion and is likely a valuable resource for services that currently support community health. We also note, however, that women were not participating in the same numbers as men in our study and that while supporting cultural diversity, informal sport does not address gender dynamics that negatively impact women’s participation in club-based sport. The qualitative data presented in the study suggest that where women did participate, they experienced similar benefits to men but our previous research has indicated that women’s participation was constrained by a range of cultural and social factors and particularly around feeling of safety as informal participants in public spaces (Jeanes *et al.*, 2019).

Thus, while informal sport represents an important avenue to support greater levels of physical activity participation across the population, addressing inequities in access to opportunities would be important in policy and resourcing efforts to support informal participation. Furthermore, our previous research has highlighted the systematic barriers informal groups face when seeking to participate, particularly around gaining access to space and facilities to play (Jeanes *et al.*, 2022).

Previous research has illustrated that informal sport is largely absent from policies aiming to support increases in sport participation or health outcomes and as a result informal sport lacks legitimacy as a format of sport participation that can contribute significantly to key policy imperatives (Jeanes *et al.*, 2022; Wheaton and O’Loughlin, 2017). To fully leverage the capability of informal sport as a health promotion resource, it is essential that informal participation is recognized as a legitimate and valuable form of sport within health promotion and sport policies, investment strategies and planning frameworks. Our data have repeatedly pointed to the need for explicit support from key stakeholders particularly local government and sporting associations, in order for health benefits to be leveraged from informal sport. Such support includes facilitating regular access to spaces to play, providing equipment and supporting infrastructure (including lights, toilets at facilities). The importance of cross-agency collaboration is also apparent, with informal sport shown to be integral to the advancement of health and community development agendas.

## LIMITATIONS

It should be noted that there are a number of limitations connected to each aspect of data collection. Despite the assumptions and limitations raised through the use of direct observation (SOPARC), these data present clear advantages over alternatives such as self-report data, particularly due to its capacity to mitigate against social desirability and recall inaccuracies. The comparative validity and reliability of SOPARC in public park and recreational spaces has been well established (McKenzie *et al*., 2006).

From the social connection survey data, it isn’t possible to ascertain how informal sport has impacted levels of social connection for individuals, we can only compare this to those participating in other forms of sport but we cannot determine if participants had high levels of social connection prior to engaging with informal sport. The mixed-method approach is valuable here however, in that interviews and focus group data did provide greater detail on the ways in which informal participation had facilitated social connections that were not readily available in other aspects of participants’ lives. Similarly, drawing on the interview and focus group data we have drawn conclusions regarding the impact of informal sport on participants’ mental health which is self-reported data rather than validated measurement of participants’ mental health. We would argue that participant perceptions still provide valuable insights into the contribution of informal sport to various dimensions of health.

## CONCLUSION

To conclude, the findings presented in the article underscore the importance of informal sport in facilitating opportunities for physical activity, fostering social connection and supporting mental health. The economic and health benefits, alongside the social and cultural value of informal sport, present valuable evidence of the need to recognize and support informal participation within communities. By leveraging existing infrastructure and the self-organizing aspects of the informal sport, local government and health stakeholders can harness its potential to improve public health outcomes and address health inequities.
